# Biophotonics in veterinary medicine: current clinical applications, translational challenges, and emerging opportunities

**DOI:** 10.3389/fvets.2026.1758597

**Published:** 2026-09-17

**Authors:** Daira Viškere, Ilze Matise-VanHoutan, Blaz Cugmas, Mindaugas Tamosiunas

**Affiliations:** 1 Preclinical Institute, Faculty of Veterinary Medicine, Latvia University of Life Sciences and Technologies, Jelgava, Latvia; 2 Biophotonics Laboratory, Institute of Atomic Physics and Spectroscopy, University of Latvia, Riga, Latvia

**Keywords:** biophotonics, imaging, optical techniques, spectroscopy, veterinary medicine

## Abstract

Biophotonics, a relatively new scientific field, applies optical or light-based techniques to biology and medicine. These approaches enable non-invasive visualization, monitoring, and characterization of biological tissues and processes, thereby supporting diagnostics, treatments, monitoring, imaging, and surgery. Biophotonic capabilities could be particularly valuable in veterinary medicine, where patients cannot verbally describe symptoms and where rapid, non-invasive assessment may reduce reliance on invasive sampling and, in selected cases, sedation or anesthesia. This perspective review examines the emerging application of biophotonics in veterinary medicine, including optical coherence tomography (OCT), fluorescence-guided imaging, hyperspectral and multispectral imaging, Raman spectroscopy, visible and near-infrared spectroscopy, and diffuse reflectance spectroscopy. Among currently available biophotonic approaches, OCT and fluorescence-guided imaging have achieved the highest level of clinical translation in veterinary medicine, particularly in ophthalmology (retinal layering assessment) and surgical oncology (real-time intraoperative tumor margin delineation). In contrast, hyperspectral imaging and Raman spectroscopy remain largely confined to research and early translational settings despite promising diagnostic performance. Across modalities, major barriers to wider clinical adoption include high equipment costs, lack of standardized protocols, and limited multicenter validation. Finally, animal patients vary considerably in haircoat, pigmentation, and skin thickness, which can affect light penetration and optical signal quality. Nevertheless, multimodal and AI-assisted approaches show strong potential to improve non-invasive diagnostics, intraoperative guidance, and physiologic monitoring in veterinary medicine.

## 1 Introduction

Despite the widespread use of conventional diagnostic modalities such as ultrasound, CT, and MRI, significant gaps remain in routine veterinary practice. While these tools excel at providing anatomical overviews, they may not provide sufficient microstructural resolution necessary to detect cellular-level changes or early-stage pathologies. Furthermore, during surgical procedures, clinicians frequently rely on subjective visual and palpatory assessments to identify diseased tissue, which can lead to incomplete resections or unnecessary damage to healthy structures.

Biophotonics offers rapid, non-contact measurement of living cells and tissues, (semi-)real-time data, and ultrasensitive detection down to single molecules across a range of temporal and spatial resolutions (1). Thus, biophotonics technologies address these limitations by serving as a crucial adjunct, providing real-time biochemical feedback and high-resolution functional data that enable more precise, objective clinical decision-making.

While biophotonics has been predominantly applied in human medicine (2, 3), several optical techniques have recently been introduced to veterinary research and practice (4–9). This paper reviews studies that have applied biophotonics to veterinary medicine to improve animal health, and highlights future prospects and untapped opportunities.

The field of biophotonics is multidisciplinary, existing at the intersection of physics, biology, chemistry, medicine, and engineering (10–12). It integrates photonics and laser technologies, biotechnologies, nanotechnologies, and the recent advancements of artificial intelligence (13–16).

Clinical biophotonics broadly encompasses diagnosis, therapy, and monitoring. This review focuses specifically on optical imaging and spectroscopic methods used for diagnosis, physiological monitoring, and intraoperative guidance; therapeutic laser-based approaches are outside its scope.

A structured literature search was conducted in Scopus, Web of Science, and Google Scholar for articles published through 01/12/2025. Search terms included: “biophotonics”, “veterinary medicine”, “spectroscopy”, “optical imaging”, “optical coherence tomography”, “OCT”, “Raman”, “surface-enhanced Raman”, “SERS”, “visible and near-infrared spectroscopy”, “VNIRS”, “hyperspectral imaging”, “multispectral imaging”, “HSI”, “MSI”, and “fluorescence imaging” (combined using Boolean operators as appropriate). Reference lists of included articles and relevant reviews were also screened.

We grouped biophotonic techniques according to how information is acquired and presented. Imaging techniques generate spatially resolved, two-dimensional representations of tissue, where each pixel contains structural or spectral information. In contrast, spectroscopic techniques typically measure light–tissue interactions at a single point, providing detailed molecular or biochemical information without forming a full image. It should be noted that this distinction is primarily practical rather than fundamental, as several optical methods—such as Raman or diffuse reflectance spectroscopy—can be implemented in both imaging and point-based formats. Here, they are categorized according to their most common clinical use.

Table 1 provides a broader overview of selected biophotonic techniques and their translation from human to veterinary medicine. However, this review focuses specifically on optical imaging and spectroscopy techniques. Other relevant biophotonic approaches, including microscopy-based techniques such as confocal and fluorescence microscopy, as well as therapeutic laser-based systems, are outside the scope of the present review and will be addressed in future work.

**Table 1:** Comparative overview of biophotonics techniques in human and veterinary medicine.

Biophotonics technique	Routine clinical field(s)—human	Human—first clinical intro (≈)	Human—widespread routine (≈)	Routine clinical field(s)—veterinary	Veterinary—first clinical intro (≈)	Veterinary—widespread routine (≈)	Notes
Flexible fiber-optic endoscopy	Gastroenterology, bronchoscopy, ENT, urology	1957–1960	1960s−1970s	GI endoscopy, rhinoscopy/bronchoscopy in small animals; equine endoscopy	1970s	1980s−1990s	Human tech led; rapid vet adoption through 80s−90s
Optical coherence tomography (OCT)—ophthalmology	Ophthalmology (retina, glaucoma)	1996–1999	2000s	Veterinary ophthalmology (canine/feline retina/cornea)	Early 2010s	Late 2010s−2020s in specialty	Rapid performance gains with spectral/fourier-domain systems
Intravascular OCT (IVOCT)	Interventional cardiology (PCI guidance)	2010	Mid-2010s	Rare/experimental	—	—	Primarily human cardiology; minimal veterinary use
Indocyanine-green near-infrared fluorescence imaging	Colorectal/HPB, breast SLNB, plastic/reconstructive perfusion, vascular, CABG perfusion checks	~2007–2010	2010s	Flap/perfusion assessment; SLN mapping in canine mammary tumors; parathyroid identification	Late 2010s	2020s	Multiple feasibility and early outcome studies in small animals
Photodynamic therapy (ALA-PDT, others)	Dermatology (actinic keratosis; some BCC/SCC *in situ*)	1999–2000	2000s	Oncology (e.g., feline cutaneous SCC; selected canine tumors)	2000s	2010s—niche	Evidence base growing; not yet ubiquitous
Reflectance confocal microscopy—skin	Dermatology (*in-vivo* ‘optical biopsy' at select centers)	Mid-late 2000s	2010s (tertiary centers)	Rare/experimental	—	—	Adopted in tertiary dermatology units; limited veterinary access
Near-infrared spectroscopy cerebral oximetry	Cardiac/vascular surgery, selected ICU/OR monitoring	Late 1990s–early 2000s	2000s−2010s	Limited clinical monitoring; research/teaching hospitals	2010s	2020s—limited	Evidence strongest in cardiac surgery; protocols vary
Photoacoustic (optoacoustic) imaging	Breast lesion diagnostics (adjunct to US)	2021	2020s (early adoption)	Preclinical/experimental	—	—	Clinical adoption just beginning in human imaging; veterinary use preclinical
Raman spectroscopy (incl. handheld intraoperative probes)	Neurosurgery (tumor margin assessment at selected centers); Dermatology (adjunct lesion assessment, limited)	2010s	2020s (adjunct, limited)	Pilot/limited use in neurosurgery; oncology (skin/soft tissue); infectious disease screening (e.g., leishmaniasis)	2020s	— not routine	Human intraoperative Raman accuracy shown in brain tumors; veterinary studies emerging (e.g., canine oncology, leishmaniasis)
Digital infrared thermography	Breast (adjunct only), diabetic foot screening/monitoring, vascular, pain/sports medicine, fever screening	1982—breast adjunct	2000s−2020s - monitoring contexts	Equine lameness/tendon injury, performance medicine; small-animal rehab/pain and dermatology (adjunct)	1990s—equine	2000s−2010s (equine); 2010s−2020s (small-animal adjunct)	Equine lameness use well-documented; in humans, thermography remains adjunct in breast screening and is used for diabetic foot monitoring

## 2 Imaging techniques

Optical coherence tomography (OCT) has been integrated into veterinary clinical practice, particularly in ophthalmology within referral settings, while fluorescence-based imaging (near-infrared fluorescence imaging), most commonly using indocyanine green (ICG), is applied primarily in specialized surgical and research environments, where it enables real-time intraoperative visualization of tissues (perfusion, tumor margins, etc.). Other biophotonic techniques for imaging (multispectral, photoacoustics, Raman, OCT) were tested as the case studies with animal patients or within research laboratory settings, e.g., using *ex vivo* biopsy submissions (17).

### 2.1 Optical coherence tomography (OCT)

Optical coherence tomography (OCT) is a non-invasive imaging technique that produces cross-sectional, grayscale images of tissue microstructure in real time (Figure 1), analogous to ultrasound imaging but using light instead of sound, resulting in much higher resolution but shallower penetration depth. OCT can produce “optical biopsies” of the cornea, retina, and optic nerve in many animal species. In clinical practice, OCT is primarily used to visualize tissue microstructure *in vivo*, enabling early detection of subtle pathological changes that are not visible with conventional imaging techniques. OCT is routinely clinically used by veterinary ophthalmologists in pets, horses, and birds to assess retinal layering, detect early glaucoma-related thinning, and monitor disease progression without invasive procedures, as well as in research models (rodents, primates) to diagnose disease, guide treatment, and monitor progression (18).

**Figure 1:**
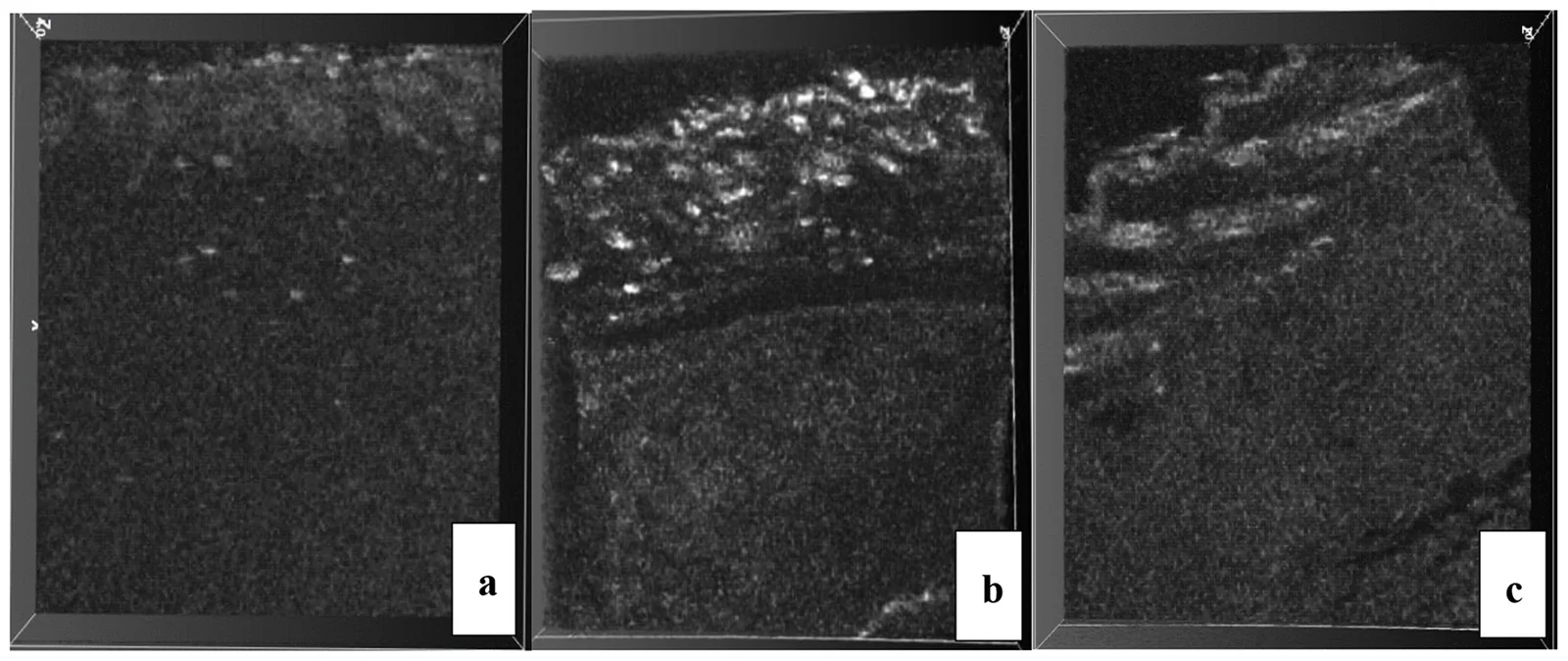
Representative optical coherence tomography (OCT) images of tumors in dogs and cats. **(a)** Lipoma, **(b)** soft tissue sarcoma, and **(c)** mast cell tumor. Images illustrate differences in tissue architecture and optical scattering patterns among the tumor types. The images were acquired using Telesto II OCT device in 1 × 1 mm scan area, and up to 2 mm penetration depth.

Technically, OCT is a depth-resolved imaging method based on low- coherence interferometry that produces micrometer-scale cross-sectional images (19). The shift from time-domain to spectral-domain OCT (SD-OCT) improved acquisition speed and motion tolerance, enabling real-time ocular imaging. Swept-source OCT provides deeper penetration, while polarization-sensitive OCT and OCT angiography (OCTA) yield functional contrasts, such as birefringence and microvascular flow.

Veterinary ophthalmology remains the most established application of OCT in practice. Since the mid-2000s, SD-OCT has been routinely applied to dogs, cats, horses, birds (20), and reptiles for high-resolution assessment of the cornea, retina, and optic nerve (21, 22). OCT enables detection of subtle changes—such as retinal thinning in glaucoma (23), photoreceptor disruption in Sudden Acquired Retinal Degeneration Syndrome (SARDS), and early stromal alterations in equine and canine keratitis—that may be missed with slit-lamp examination or ultrasonography. Species-specific normative datasets continue to expand, improving interpretation.

Beyond ophthalmology, OCT is increasingly explored in surgical oncology. Studies show clear discrimination between neoplastic and normal tissue in tumors such as feline injection-site sarcoma (24), canine mammary tumors (25), mast cell tumors (26), anal sac adenocarcinoma (5), and soft-tissue sarcomas (6, 9, 27). Handheld probes demonstrate rapid learnability; even minimally trained users can achieve sensitivity and specificity exceeding 88% and 92%, respectively, for margin discrimination, supporting real-time intraoperative use when histopathology is unavailable. However, intraoperative factors such as blood pooling, tissue desiccation, and thermal or mechanical artifacts from surgical instruments can alter OCT signals and obscure the underlying tissue architecture (8).

Additional applications include dermatology—where OCT enables rapid noninvasive assessment of superficial lesions and ectoparasites (28, 29)—and orthopedics, where arthroscopic OCT reveals microstructural cartilage abnormalities not visible with standard arthroscopy (30).

Artificial intelligence (AI) and multimodal imaging are emerging areas of focus in veterinary biophotonics. Convolutional neural networks applied to OCT images have achieved 98% sensitivity and 97% specificity for canine soft-tissue sarcoma margin detection (31). Multimodal imaging refers to the combination of two or more optical techniques to obtain complementary information—for example, pairing OCT, which provides high-resolution structural images, with Raman spectroscopy or fluorescence imaging, which reveal biochemical composition. In this combination, the approach achieved 93% sensitivity and 97% specificity across 1,000 images and spectra from 51 biopsy samples (32).

The main technology limitations include the system costs (starting at US$ 45,000) and a shallow penetration (1–3 mm), making it useful for assessing superficial and lateral margins but insufficient for evaluating deep vertical margins involving fascia or muscle. Additionally, sedation may be required in many species to obtain high-quality images, which can limit routine use in general practice. Research-wise, there is a lack of standardized protocols and bigger study populations.

Future development hinges on species-specific reference libraries, interpretable AI tools, standardized imaging protocols, and wider availability of portable devices suitable for general practice. With these advances, OCT is well-positioned as a routine adjunct in veterinary ophthalmology and surgical oncology.

### 2.2 Fluorescence-based imaging

While OCT provides depth-resolved structural information, fluorescence-based imaging adds functional and biochemical contrast by visualizing light emitted from tissues or administered dyes (Figure 2). In veterinary practice, clinicians are already familiar with the basic principle of fluorescence through the use of a Wood's lamp in dermatology, where ultraviolet (UV) light induces visible apple-green fluorescence in dermatophyte infections, such as *Microsporum canis*. However, modern fluorescence imaging in veterinary medicine extends far beyond this application. In particular, near-infrared (NIR) fluorescence imaging uses exogenous fluorophores such as indocyanine green (ICG), which emit light in the near-infrared range and allow deeper tissue visualization with reduced background interference. Unlike Wood's lamp, which is limited to superficial dermatological assessment, NIR fluorescence imaging enables real-time intraoperative visualization of tissue perfusion, lymphatic drainage, tumor margins, and anatomical structures such as bile ducts.

**Figure 2:**
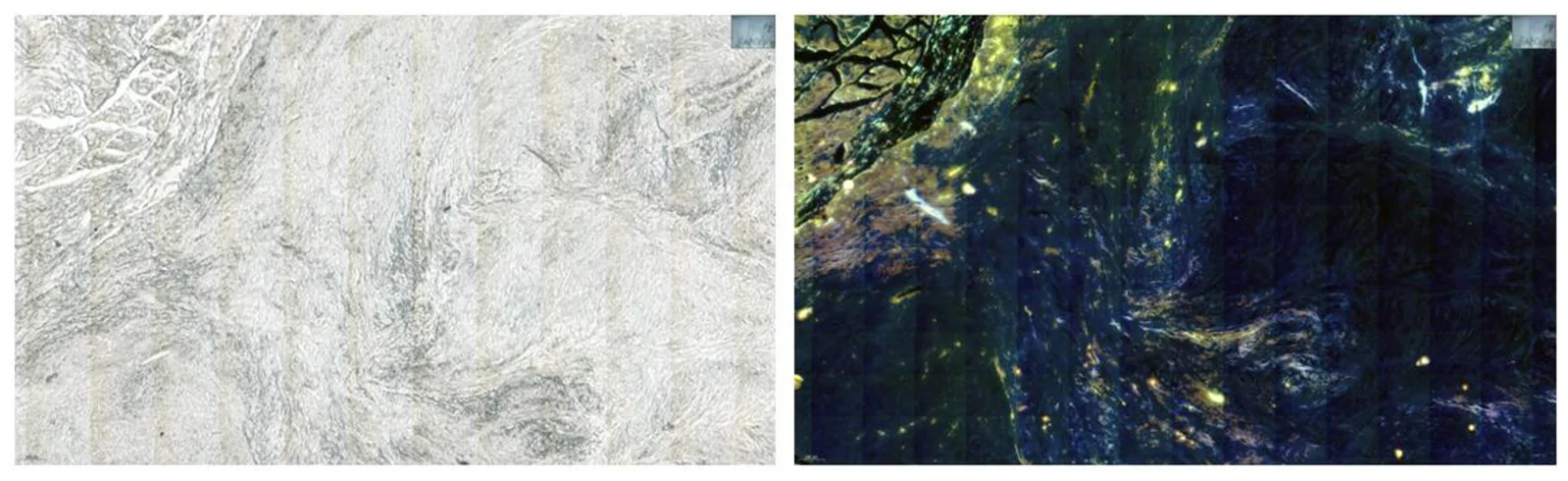
Comparison of conventional brightfield and autofluorescence imaging of an unstained canine soft tissue sarcoma histopathology slide. Left: native brightfield image acquired at 5 × magnification. Right: corresponding autofluorescence image of the same unstained section obtained using four fluorescence channels (DAPI, FITC, Cy3, and Cy5). The autofluorescence image demonstrates wavelength-dependent endogenous tissue contrast without the use of histological staining.

Technically, fluorescence imaging visualizes endogenous, i.e., naturally occurring within tissues (Figure 2) or exogenous fluorophores, externally administered, to characterize tissue structure and biochemistry. In veterinary medicine, exogenous dyes such as indocyanine green (ICG) and 5-aminolevulinic acid (ALA), are most commonly used because endogenous autofluorescence does not provide sufficient contrast. Compared to visible light (380–700 nm), near-infrared (NIR, ~700–900 nm) excitation enables deeper tissue penetration, reduced background autofluorescence, and higher signal-to-noise ratios. ICG fluorescence is the best-established intraoperative fluorescence technique in companion animal surgery. Reflecting human applications, it has been used for sentinel lymph node mapping, perfusion assessment, and tumor visualization (33–35). In dogs, ICG provides strong contrast for pulmonary adenocarcinomas and soft-tissue sarcomas (36, 37). However, nonspecific ICG accumulation may result from the enhanced permeability and retention (EPR) effect. Because inflamed or hypervascular tissues can show similar dye uptake, this may cause false-positive fluorescence and overestimation of tumor margins (36, 37). Even so, the combination of ICG with point spectroscopy accurately identified tumor margins in most canine cases.

Advanced fluorescence techniques such as fluorescence lifetime imaging (FLIm), which measures the decay time of emitted fluorescence rather than its intensity, are also being explored in veterinary medicine, including studies on spontaneous canine oral tumors, with promising translational potential (38). Hybrid fluorescence approaches and machine-learning–based interpretation are advancing rapidly. For example, combining NIR autofluorescence with Raman spectral-band imaging achieved 90% accuracy for differentiating benign and malignant soft-tissue tumors in veterinary biopsy samples (39).

Clinical implementation also depends on hardware configuration. Fluorescence-imaging systems may be gantry-mounted for open surgery, handheld for flexible assessment, or integrated into endoscopic or laparoscopic platforms. Integrated systems are particularly relevant to minimally invasive surgery (MIS), where fluorescence can be displayed alongside the standard operative image.

As with OCT, fluorescence imaging is limited by high system costs and shallow penetration (typically a few millimeters). However, unlike OCT, it does not provide depth-resolved structural information, and signal intensity depends on dye distribution, which may vary with perfusion and inflammation, potentially reducing spatial accuracy. Although ICG is generally well tolerated, rare hypersensitivity reactions, including anaphylaxis, have been reported; because it is cleared almost exclusively by the liver into bile, severe hepatic dysfunction may also alter its clearance and imaging kinetics (40). Additional limitations include variability due to pigmentation and haircoat, and limited clinical validation.

Future directions emphasize standardized imaging protocols, quantitative metrics, wider use of lifetime- and spectrally resolved fluorescence, and multimodal platforms that integrate fluorescence with OCT, Raman spectroscopy, or photoacoustic imaging. AI-based interpretation and larger clinical datasets will be critical for achieving reliable intraoperative guidance.

### 2.3 Hyperspectral and multispectral imaging (HSI/MSI)

Beyond structural and fluorescence-based imaging, spectral imaging techniques such as HSI and MSI capture wavelength-dependent tissue information over a wider field of view (Figure 3). Spectral imaging can be understood as an extension of standard digital imaging, in which a conventional camera records three broad color channels: red, green, and blue (RGB). Multispectral imaging expands this approach by capturing a small number (up to 10) of additional wavelength-specific images beyond RGB, while hyperspectral imaging acquires a much larger number (>10) of narrow wavelength bands, generating a detailed spectral profile for each pixel (41).

**Figure 3:**
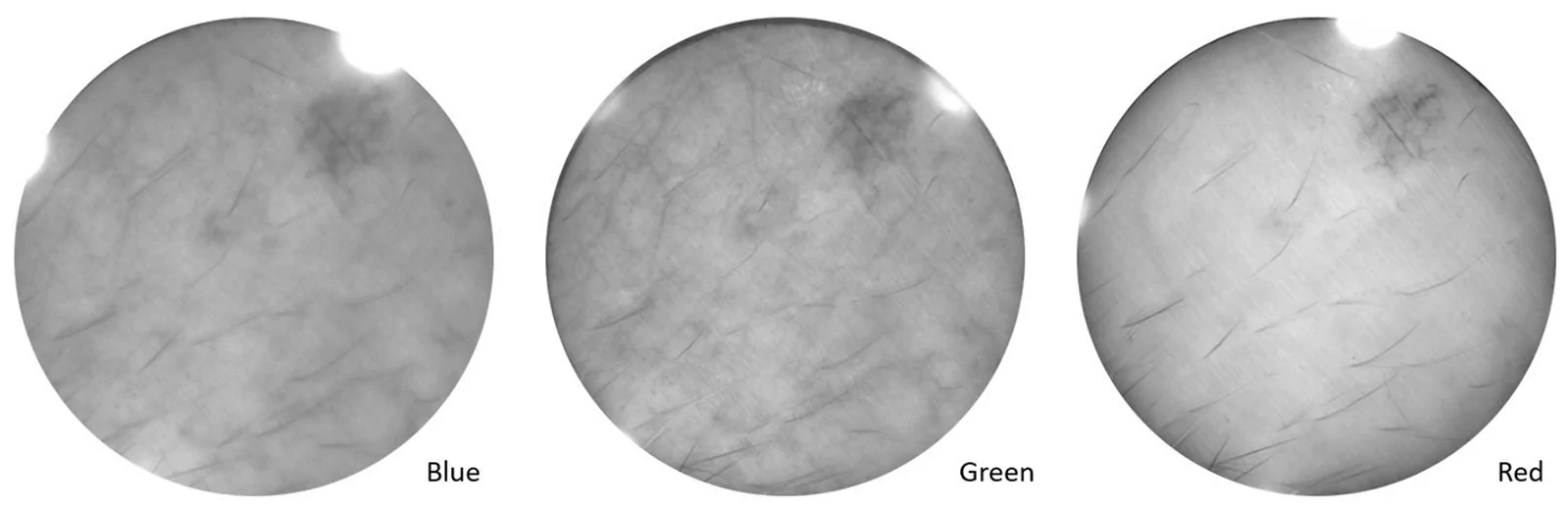
Representative multispectral dermatoscopic images of canine skin acquired at different visible wavelengths (blue, green, and red). Shorter wavelengths (blue) emphasize superficial skin structures, whereas longer wavelengths (red) provide enhanced visualization of deeper tissue features.

Spectral information allows clinicians to assess tissue composition and physiology, including oxygenation, blood content, hydration, and inflammation. As a result, HSI and MSI can provide functional information that is not visible in standard imaging, supporting applications such as perfusion assessment, wound evaluation, and tumor detection. In comparison, HSI offers richer biochemical discrimination, while MSI provides faster acquisition and simpler hardware, which is highly advantageous in field or clinical settings. Spectral interactions—including wavelength-dependent absorption, scattering, and fluorescence—enable assessment of hemoglobin oxygenation, hydration, lipid content, and inflammation (42).

In human medicine, HSI/MSI is widely used for wound assessment, flap viability, ischemia detection, dermatologic evaluation, and intraoperative perfusion mapping (43, 44). Veterinary applications are still in the early stages, but promising.

Studies demonstrate utility for tumor-margin delineation in canine and feline oncology (44, 45), wound or graft monitoring in small animals, and early perfusion assessment in equine limb conditions. In production animals, spectral imaging has aided in detecting feed contamination (36, 46) and assessing poultry health (47). HSI is preferable when fine biochemical discrimination is needed, while MSI is better suited for rapid triage, equine sports medicine, and livestock monitoring. For serial wound assessment or emergency care, MSI provides adequate contrast with minimal workflow disruption.

Compared to OCT and fluorescence imaging, MSI/HIS typically exhibit deeper tissue visualization, as longer wavelengths (particularly in the red and near-infrared range) penetrate further into tissue (several milimeters). However, signal quality remains highly sensitive to haircoat, pigmentation, moisture, ambient light, and probe-to-tissue distance. For example, high melanin concentration in dark-furred breeds or pigmented tissues can strongly absorb light, potentially masking underlying hemoglobin or other biochemical spectral signatures and complicating accurate margin delineation in veterinary oncology patients.

A key limitation is also the lack of standardization—spectral signatures vary across species, anatomical sites, and devices, making it difficult to transfer models between different clinical applications. As a result, many systems remain application-specific, and findings from one setting may not generalize to others. Moreover, in field settings such as open barns or paddocks, ambient light can interfere with spectral measurements, requiring light-shielding accessories or ambient-light correction methods to improve reliability. Finally, most veterinary studies rely on small, single-center datasets, which limit reproducibility and increase the risk of overfitting in machine-learning approaches.

Combining HSI/MSI with thermography, ultrasound, fluorescence, or OCT may reduce false positives and provide complementary structural and biochemical information. Portable, handheld systems are emerging for sports medicine, field anesthesia, wound care, and livestock health. Clinical translation will require standardized acquisition protocols, normalization strategies, and multicenter validation. As hardware becomes more compact and analysis more automated, HSI/MSI are likely to serve as adjuncts for perfusion assessment, wound evaluation, early inflammation detection, and surgical decision support in veterinary practice.

## 3 Spectroscopy techniques

In contrast to imaging techniques, which provide spatially resolved two-dimensional information (image), spectroscopic approaches focus on point-based measurements, acquiring detailed optical signatures from a localized region of tissue. These techniques probe molecular composition through characteristic absorption, scattering, or vibrational features, offering highly specific biochemical information. While many spectroscopic methods can also be extended to imaging modalities, their point-based implementations remain the most widely used in veterinary applications, particularly for rapid diagnostics and tissue characterization.

### 3.1 Raman spectroscopy

Raman spectroscopy is an optical technique based on light scattering, in which a small fraction of the incident light interacts with molecular vibrations in tissue and is scattered with a slight shift in wavelength (48). This shift provides a characteristic ‘molecular fingerprint', allowing identification of biochemical components such as proteins, lipids, nucleic acids, and metabolites without the need for external dyes (49, 50). Unlike absorption-based methods, Raman spectroscopy is highly specific to molecular structure. In clinical terms, Raman spectroscopy can be used to distinguish between healthy and diseased tissue based on their biochemical composition, for example, to differentiate benign from malignant tumors.

The Raman signal is inherently very weak—typically several orders of magnitude lower than the incident light—and can be easily influenced or obscured by background signals such as fluorescence (Figure 4). As a result, measurements often require careful signal processing, longer acquisition times, or signal enhancement techniques. However, Raman preprocessing methods, including baseline correction and polynomial fitting, are not yet standardized, and methodological differences may alter peak intensities and limit the transferability of machine-learning models across instruments or centers.

**Figure 4:**
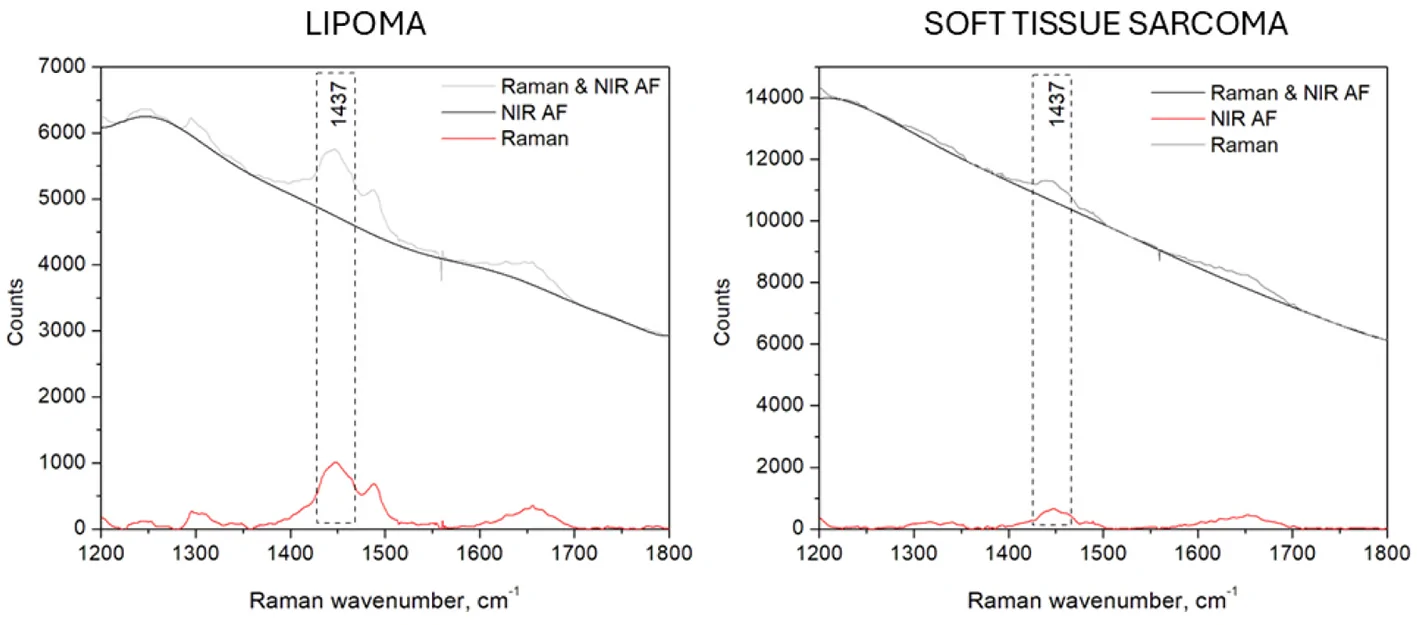
Representative Raman spectroscopy measurements of canine adipose and soft tissue tumors. Left: Raman spectrum acquired from a canine lipoma. The upper spectrum shows the combined near-infrared autofluorescence and Raman signal, while the lower spectrum demonstrates the isolated Raman spectrum after subtraction of the autofluorescence background (NIR AF). A prominent Raman band at approximately 1,437 cm^−1^, associated with lipid-rich tissue, is clearly visible and reflects the high fat content characteristic of lipoma. Right: Representative Raman spectrum of canine soft tissue sarcoma, in which the 1,437 cm^−1^ lipid-associated band is less pronounced, demonstrating differences in biochemical composition between adipose and non-adipose tumors.

Advances in compact spectrometers, fiber-optic probes, and surface-enhanced Raman spectroscopy (SERS) have expanded veterinary applications. Raman spectroscopy has shown promising diagnostic performance in veterinary oncology, with studies of canine cancer detection and tumor discrimination (Figure 4) reporting sensitivity and specificity values around 90% (32, 39, 51–53). Related human and methodological studies further support the translational potential of Raman-based and multimodal classification approaches (32, 39, 49, 54). Clinical use of intraoperative fiber-optic probes also requires careful sterility management, as many probes are not compatible with autoclaving and may require low-temperature sterilization or disposable sterile sheaths, which can attenuate the optical signal or introduce artifacts. Moreover, routine SERS screening is limited by the recurring cost of disposable gold- or silver-based substrates and batch-to-batch variability in signal enhancement, which complicates calibration and reproducibility.

Raman also supports infectious disease detection, identifying *Leishmania, Babesia bovis*, fish pathogens, ticks, and mastitis-associated bacteria (55–57). Raman-based metabolic profiling detects exercise- associated biochemical shifts in horses (58, 59) and inflammation-related changes in rodent models. Additional applications include species identification for wildlife forensics and food authentication (60). SERS platforms are increasingly used for rapid detection of drug residues in milk, meat, and aquaculture products (61). Machine learning—ranging from classical chemometrics (PCA, LDA, SVMs) to deep neural networks—has been transformative. Multimodal Raman–OCT or Raman–autofluorescence combinations further enhance diagnostic accuracy (32, 62).

In biological tissues, Raman signals are often further obscured by background fluorescence, which can dominate the detected signal, particularly in pigmented or inflamed tissues. There is also variability in the signal due to species-specific anatomy and instrument design. Small datasets and a lack of standardization limit generalizability, and portability and cost remain barriers in field settings.

Future progress requires improved signal-to-noise ratios, optimized SERS substrates, species- specific spectral libraries, and multicenter validation. Integration with OCT, FLIm, and spectral imaging may provide complementary biochemical and structural information, advancing clinical utility.

### 3.2 Visible and near-infrared spectroscopy (VNIRS)

Visible and near-infrared spectroscopy (VNIRS) is conceptually based on the same optical principles as spectral imaging techniques, in that it analyzes how tissue interacts with light across a range of wavelengths. However, instead of generating a spatial image, VNIRS acquires a spectral signal from a localized area, measuring tissue absorption, scattering, and reflectance across the 400–2,500 nm range. This provides rapid insight into physiological parameters such as oxygenation, perfusion, hydration, and metabolic state (63, 64). VNIRS generates spectral curves from a small interrogated area, offering real-time, noninvasive assessments with relatively simple and portable hardware.

Because diffuse reflectance spectroscopy (DRS) is closely related to VNIRS, it is included in this section rather than discussed separately. DRS can be considered a reflectance-based implementation of visible and near-infrared spectroscopy, in which diffusely reflected light from tissue is collected and analyzed to estimate tissue composition and physiological parameters (Figure 5). This approach is commonly used for point-based assessment of chromophores such as hemoglobin, melanin, water, and lipids, as well as scattering-related changes associated with tissue structure (2, 65).

**Figure 5:**
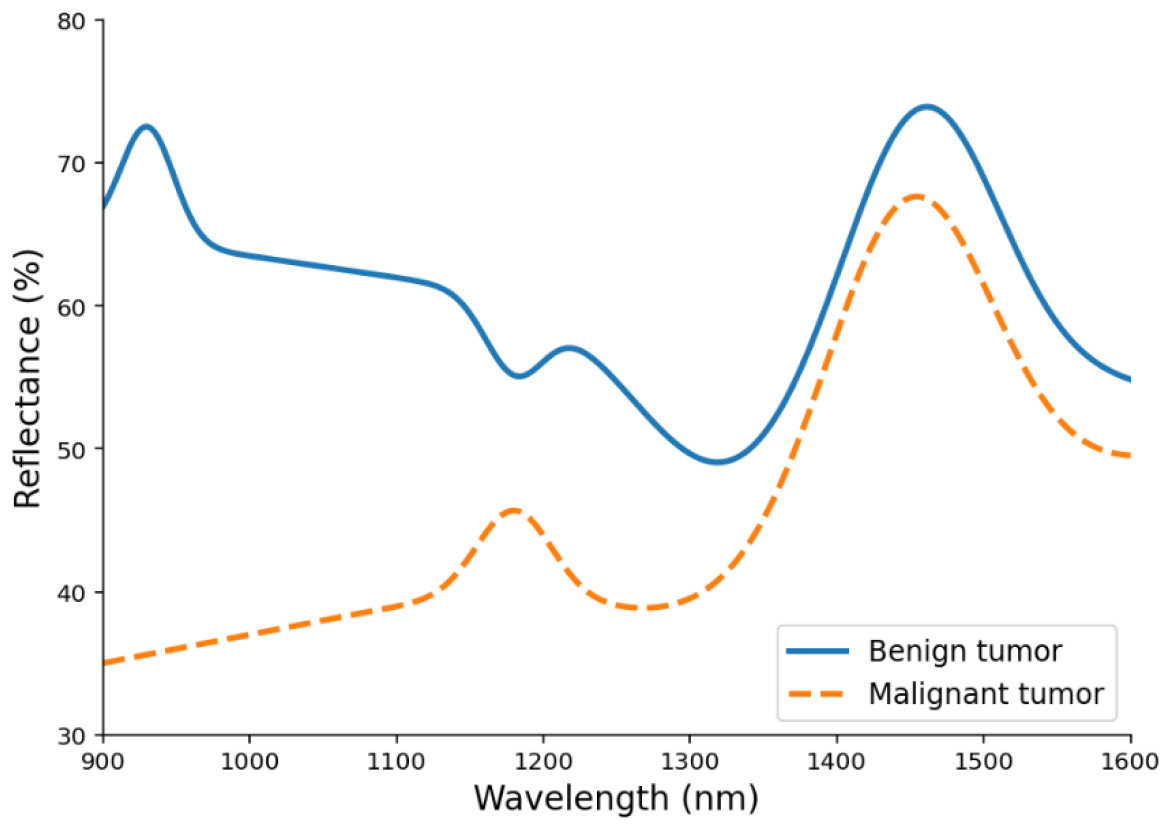
Representative diffuse reflectance near-infrared spectra illustrating differences in wavelength-dependent reflectance between benign and malignant tumors. Variations in spectral shape and intensity reflect differences in tissue composition, vascularization, scattering, and biochemical properties.

VNIRS has been explored in human medicine for tumor discrimination, perfusion assessment, and biopsy guidance (66). Veterinary studies remain early-stage but indicate value for characterizing skin lesions, assessing wound viability, differentiating inflamed from healthy tissue, and evaluating superficial masses in dogs, cats, horses, and livestock. Sensitivity to oxygenation and blood volume makes DRS promising for mastitis monitoring in dairy cattle (67) and for early ischemia detection in equine limbs (68), which could be done at relatively superficial sites such as the coronary band or over the superficial digital arteries. Its low cost and portability are appealing for field or farm applications.

VNIRS has also shown potential for identifying early biochemical changes in oral or cutaneous tumors (38). In farm settings, portable devices support feed-quality assessment, hay moisture and protein measurement, and early mycotoxin detection (69, 70).

VNIRS is appealing because it provides continuous physiological information without the need for sedation or invasive sampling. Shifts in hemoglobin absorption peaks can reveal early ischemia or metabolic changes, enabling earlier clinical decisions.

Penetration depth in VNIRS is wavelength-dependent: shorter wavelengths (blue/visible light) probe superficial layers, while red and near-infrared light can penetrate more deeply into tissue, although still limited by scattering. However, measurements remain highly sensitive to pigmentation, haircoat, probe pressure, hydration, ambient light, and motion. In particular, probe pressure can alter local blood flow and tissue optical properties, directly affecting the measured spectrum (71). Additionally, overlapping spectral signatures across different conditions require robust reference datasets and advanced analytical methods, while inter-instrument variability further complicates reproducibility, as models developed on one device may not perform reliably on another because of differences in light sources, detectors, and spectrometer sensitivity.

Recent research has focused on improved signal processing, portable devices, and multimodal platforms integrating VNIRS with thermography, ultrasound, Raman spectroscopy, OCT, or fluorescence. Machine-learning methods (SVMs, logistic regression, deep neural networks) have improved VNIRS-based detection of mastitis, metabolic disorders, and tumor-associated changes (65, 72).

VNIRS is likely to find its most practical role in targeted applications such as perioperative monitoring, triage, metabolic screening, and on-farm quality control.

Limitations of VNIRS/DRS include sensitivity to pigmentation, hair coat, hydration, ambient light, motion, probe-to-tissue distance, and, especially, probe pressure, which can alter local perfusion and the measured spectrum. Penetration depth is wavelength-dependent, with red and near-infrared light probing deeper than shorter visible wavelengths, but measurements remain strongly influenced by scattering and superficial tissue properties. Lack of standardized protocols, species-specific spectral libraries, and robust validation limits reproducibility and clinical translation.

Future progress should focus on standardized acquisition, correction for pigmentation and hair coat effects, larger reference datasets, and portable devices. VNIRS/DRS will likely be most useful as an adjunct for perfusion monitoring, wound assessment, triage, and on-farm screening rather than as a standalone diagnostic tool.

## 4 Conclusions and future perspectives

Biophotonics comprises complementary modalities that can materially improve veterinary diagnosis. Each technique brings distinct strengths; chemistry-sensitive methods (Raman, VNIRS, DRS) quantify molecular composition, while structure-focused techniques (OCT, HSI) resolve microanatomy. Surface tools (MSI/HSI, VNIRS) enable rapid, low-stress screening but are limited by environment and depth. On the other hand, depth-resolving methods (OCT, fiber-optic Raman) provide richer information at a higher cost and with greater skill requirements, and sometimes require sedation.

To support clinical comparison, Table 2 summarizes the main veterinary applications, species examples, current implementation status, representative equipment, and approximate cost ranges of the reviewed biophotonic techniques.

**Table 2:** Biophotonic diagnostic and imaging techniques in veterinary medicine regarding the application.

Diagnostic and imaging technique/method	Main application(s)	Species examples	Status	Representative equipment	Price range
Optical coherence tomography (OCT)	High-resolution corneal/retinal imaging; evaluation of skin lesions; intraoperative tumor margin assessment	Dogs, cats, horses, birds, reptiles	Specialty clinic	Heidelberg Spectralis OCT2; Optovue iVue; Zeiss Cirrus HD-OCT	$45,000–$120,000
Fluorescence-based imaging (ICG, ALA, autofluorescence, FLIm)	Perfusion mapping, sentinel node guidance, tumor visualization, intraoperative assessment	Dogs/cats, horses, rodents	Specialty clinic/emerging clinical	Fluobeam LX; VisionSense NIR; PDE-Neo II; FLIm fiber systems	$30,000–$150,000 (FLIm $80k–$200k)
Endoscopic fluorescence imaging	GI/airway visualization, mucosal lesion enhancement, perfusion mapping	Dogs, cats, horses, camelids	Specialty clinic	Karl Storz IMAGE1 S NIR/ICG; Olympus Visera Elite II	$60,000–$180,000
Hyperspectral and multispectral imaging (HSI/MSI)	Perfusion/oxygenation assessment, ischemia detection, wound monitoring	Horses, dogs/cats, cattle, poultry	Limited clinical/research	Cubert Univisio HSI; Specim IQ; TIVITA tissue	$15,000–$80,000
Near-infrared spectroscopy (NIRS/VNIRS)	Regional oxygenation monitoring, perfusion assessment	Foals, horses, neonatal dogs/cats, rodents	Limited clinical	Masimo root O3; INVOS 5100C; artinis PortaMon/PortaLite	$10,000–$45,000
Diffuse reflectance spectroscopy (DRS)	Perfusion, oxygenation, superficial tumor or wound assessment	Dogs/cats, horses, cattle	Limited clinical/research	Verisante aura; ocean insight FLAME/FX DRS probes	$5,000–$30,000
Raman spectroscopy	Tumor discrimination, infectious disease detection, metabolic profiling, food safety	Dogs, cats, horses, cattle, rodents	Experimental to early clinical	B&W Tek i-Raman Plus; Renishaw inVia; Agilent Resolve	$25,000–$150,000

### 4.1 Limitations and standardization

Species-specific and contextual factors must be considered, as optical signals are influenced by coat, pigmentation, skin thickness, and anatomy, as well as behavioral constraints such as the need for sedation. Examples include coat-dependent variability in livestock imaging and sedation requirements for equine ocular studies, necessitating species-specific calibration and the inclusion of diverse breeds in validation cohorts. Discussion of safety issues is imperative, particularly regarding sedation risks that impact animal welfare, as optical tools may reduce invasive sampling but still require restraint.

Not all optical methods are equivalent; non-invasive surface tools are efficient but limited in depth sensitivity, while imaging techniques offer greater detail at the cost of higher hardware and operational demands. It is vital to clarify this taxonomy to help clinicians match tools to specific clinical issues.

Translation also relies on strong standardization and empirical evidence, and requires multicenter, adequately powered studies that avoid overfitting and prioritize harmonized acquisition and reporting. At the moment, the lack of standardization is one of the main challenges, as current high-accuracy claims are based on small, single-center cohorts that may include overfitted models. Thus, multicenter validation studies with standardized protocols and publicly accessible spectral/image libraries are crucial. Publicly accessible spectral and image datasets could be shared through open repositories such as Zenodo, while a dedicated veterinary biophotonics working group could coordinate harmonized acquisition protocols, metadata standards, and multicenter data sharing.

Additionally, regulatory clarity and quality control are essential, encompassing device calibration, dosimetry, and operator competency.

### 4.2 Economic consideration

Based on our experience, equipment costs are among the main reasons for the slow clinical translation of biophotonics. However, economic considerations extend beyond equipment costs to include maintenance, disposables, cloud analytics, and training where additional operational barriers include limited or inconsistent insurance reimbursement for advanced optical procedures and the time and training required for clinicians to interpret complex spectral or tomographic data. Cost-effectiveness studies are recommended, such as assessing whether intraoperative OCT justifies its purchase by reducing re-excision rates, and emphasizing the importance of low-cost, ruggedized devices for field use.

### 4.3 Multimodal approaches

The literature frequently indicates that multimodal approaches are the most effective, as strategies combining techniques such as OCT and Raman, or OCT and thermal/DRS, yield superior results for tumor discrimination and margin assessment by integrating structural and chemical contrasts.

### 4.4 Future perspectives

Among biophotonic approaches, high-resolution label-free modalities (OCT, HSI) and targeted fluorescence imaging show the greatest near-term promise for clinical translation. On the other hand, Raman (e.g., SERS) and diffuse optical techniques remain more challenging due to weak signals, hardware complexity, and demanding data analysis.

Optical technologies are unlikely to replace established clinical methods, but they are well positioned to support routine triage, intraoperative decision-making, and non-invasive monitoring across veterinary specialties. With appropriate modality selection, non-contact surface techniques may reduce the need for sedation or anesthesia during routine assessment, whereas higher-resolution modalities may be most useful for intraoperative guidance in patients already under anesthesia. Successful clinical translation will depend on targeted validation, standardized protocols, species-aware calibration, and practical integration into veterinary workflows.

## Data Availability

The original contributions presented in the study are included in the article/supplementary material, further inquiries can be directed to the corresponding author.
